# EFFECT OF ACA MEDICAID EXPANSION ON THE LABOR SUPPLY OF DIRECT CARE WORKERS

**DOI:** 10.1093/geroni/igac059.1137

**Published:** 2022-12-20

**Authors:** Lili Xu, Hari Sharma

**Affiliations:** University of Iowa, Iowa City, Iowa, United States; University of Iowa, Iowa City, Iowa, United States

## Abstract

Direct care workers (DCWs) such as personal care aides, home health aides, and nursing assistants provide critical care to patients and residents in different settings including at home, nursing homes, and hospitals but DCWs earn low wages with limited benefits. The Affordable Care Act Medicaid expansion increased health insurance access among low-income individuals but there are concerns that public insurance may disincentivize labor supply. In this study, we examine whether Medicaid expansion affected the labor supply of low-educated DCWs at both extensive and intensive margin overall, and by different healthcare settings. Using annual American Community Survey data from 2010 to 2019 retrieved via Integrated Public Use Microdata Series, we identify 100,676 adult DCWs (age: 19-64) with a high school or less degree from 50 states and DC. We examine the potentially causal effect of Medicaid expansion on labor supply of DCWs using difference-in-differences and event-study regressions We find that Medicaid expansion is associated with a 2.9 percentage-point (p< 0.01) increase in full-time employment (>=35 hours) and a 1.9 percentage point (p< 0.05) decrease in part-time employment (20-34 hours). We also find that unemployment decreased by 0.8 percentage points (p< 0.1) among DCWs mainly driven by those working in the long-term care industry. Our study suggests that Medicaid expansion does not have a negative impact on labor supply among low-educated DCWs. States that have not expanded Medicaid can consider policies to increase insurance coverage for DCWs as a strategy to strengthen this workforce.

